# Novel Biomarkers in Pancreatic Cancer

**DOI:** 10.3390/cancers16030628

**Published:** 2024-01-31

**Authors:** Alessandro Coppola, Daniela Pozzi, Damiano Caputo

**Affiliations:** 1 Department of Surgery, Sapienza University of Rome, Via Giovanni Maria Lancisi 2, 00161 Rome, Italy; coppola.chirurgia@gmail.com; 2 NanoDelivery Lab, Department of Molecular Medicine, Sapienza University of Rome, Viale Regina Elena 291, 00161 Rome, Italy; 3 Fondazione Policlinico Universitario Campus Bio-Medico, Via Alvaro del Portillo 200, 00128 Rome, Italy; d.caputo@policlinicocampus.it; 4 Research Unit of Surgery, Department of Medicine and Surgery, University Campus Bio-Medico di Roma, Via Alvaro del Portillo 21, 00128 Rome, Italy

Pancreatic ductal adenocarcinoma (PDAC) represents a neoplasm with an increasing incidence in both sexes. Unfortunately, in contrast to what has been observed in other cancers, the mortality rate for PDAC remains high [1].

What is currently evident is our inability to diagnose the tumor at its earliest stages or even in a pre-tumoral state, as we do, for instance, with colon adenocarcinoma. This is mainly due to the lack of large-scale effective screening methods. Therefore, the identification of blood serum markers able to ensure an early diagnosis of PDAC is crucial. Moreover, in the case of PDAC diagnosis, markers can also play a crucial role in staging the disease accurately, identifying the correct therapeutic pathway, and monitoring these patients.

Currently, the only marker approved for clinical use for PDAC management is carbohydrate antigen 19.9 (CA 19.9). Unfortunately, this marker alone cannot meet all the abovementioned requirements. Due to its low sensitivity and specificity, CA 19.9 alone cannot be used as a screening tool for the early diagnosis of PDAC.

For this reason, there are studies in the literature which have investigated the role of different protein markers and pancreas-specific cell-free DNA (cfDNA) methylation that, in association with CA 19.9, could increase the ability to diagnose PDAC early [2].

Unfortunately, despite these promising results, these marker panels cannot be used in daily clinical practice as they do not fully meet the practicality, reproducibility, and cost containment criteria required by the World Health Organization for a marker [3].

To fill this gap, nanotechnologies have been successfully tested to identify biological markers and signatures capable of enabling early diagnosis in patients with PDAC. Nanotechnologies, either alone or in combination with other inflammatory markers, have proved their ability to contribute to a more accurate staging of PDAC. This approach holds the promise of paving the way for generating personalized therapies for each individual patient [4].

Unfortunately, work in this field is still in the research phase, and additional larger confirmation studies are needed before any findings can be translated into routine clinical practice.

In recent years, CA 19.9 has played a significant role in improving the staging and follow-up of patients undergoing surgical treatment or receiving chemotherapy or radiotherapy for PDAC.

Indeed, the growing concept of biological staging has led to patients with PDAC initially considered radiologically resectable being reclassified as borderline resectable due to elevated levels of CA 19.9. This concept was well defined during the consensus meeting of the International Association of Pancreatology (IAP), which considered marker values in addition to anatomical factors in staging [5].

All of this is not without therapeutic and, above all, clinical impact. Patients are more readily referred to neoadjuvant treatments, and the changing marker values play a prognostic role in these patients [6,7]. 

Patients with PDAC and elevated CA 19.9 levels have also been found to be at a higher risk of lymph node metastasis. This aspect is crucial because lymph node involvement significantly alters prognosis for these patients. In a recent study by Stobel, nodal positivity correlated with worse overall survival. Additionally, patients without lymph node involvement and normal CA 19.9 levels at diagnosis were the only ones to show 5-year disease-free survival curves after surgical intervention [8].

The relationship between lymph node positivity and marker values was well demonstrated in a recent multicenter international retrospective study, highlighting that over 80% of patients with elevated CA 19.9 levels have lymph node metastases even in conditions of anatomical resectability [9].

The issue of patients in whom CA 19.9 is not expressed is certainly one of the limitations of the previous study and, in general, the use of CA 19.9 in the staging of PDAC. Consequently, the decision making for the treatment of these patients often turns out to be inadequate. In this regard, efforts are being made to identify markers that, in addition to CA 19.9 or in cases where CA 19.9 is not expressed, can assist in the accurate staging and treatment of these patients.

A recent study by Doppenberg demonstrated how carcinoembryonic antigen (CEA) can be helpful in patients with negative CA 19.9 values (i.e., <37 U/mL). In this study, localized PDAC patients with elevated CEA levels at both diagnosis and after neoadjuvant treatment with Folfirinox were associated with a statistically significant worsening of overall survival, from 33 months to 19 months. Therefore, as suggested by the authors, in cases of CA 19.9 negativity, CEA levels can certainly be a helpful tool to use in localized PDAC patients [10].

Another marker used in the place of CA 19.9 in non-secretory patients is the Duke Pancreatic Monoclonal Antigen Type 2 (DUPAN-2). Omiya et al. demonstrated that, in their study, the survival curves for patients who are non-secretors of CA 19.9 but have DUPAN-2 levels >2000 U/mL were similar to those of patients with CA 19.9 values >500 U/mL [11].

In other models, combinations of all three markers, CA 19.9, CEA, and DUPAN-2, are proposed either before or after neoadjuvant treatments to better stage the prognosis of PDAC patients [12].

In patients expressing CA 19.9, the marker proves to be crucial in post-surgical follow-up. It is widely reported that a new elevation of the marker should be considered as a recurrence of the disease even if not yet evident in radiological examinations [13].

The failure of CA 19.9 to normalize after surgery can also be an indicator of an unfavorable prognosis in patients treated with certain chemotherapy regimens. Therefore, in these patients, more aggressive adjuvant therapies than those currently used in the majority of patients may be recommended [14].

In the fight against PDAC, neoplastic markers are one of the most active and promising research fields. Currently, the only recognized marker is CA 19.9, which can be used either alone or in combination with other markers. The discovery of a new marker or the right combination of existing markers could represent a significant step forward in PDAC treatment. One aspect that makes markers particularly appealing is that marker values can be standardized on a scale and reproducible in any laboratory, allowing for rapid clinical application worldwide.

## Data Availability

Not applicable.
